# Supplementary material for: Time-invariant working memory representations in the presence of code-morphing in the lateral prefrontal cortex
Source: Nat Commun. 2019 Nov 1;10:4995. doi: 10.1038/s41467-019-12841-y (PMC6825148; doi:10.1038/s41467-019-12841-y)
Supplement: Supplementary file 1 — Supplementary Information [file 41467_2019_12841_MOESM1_ESM.pdf]

Time-Invariant Working Memory Representations in the Presence of Code-Morphing in the Lateral Prefrontal Cortex
Parthasarathy, *et al*.

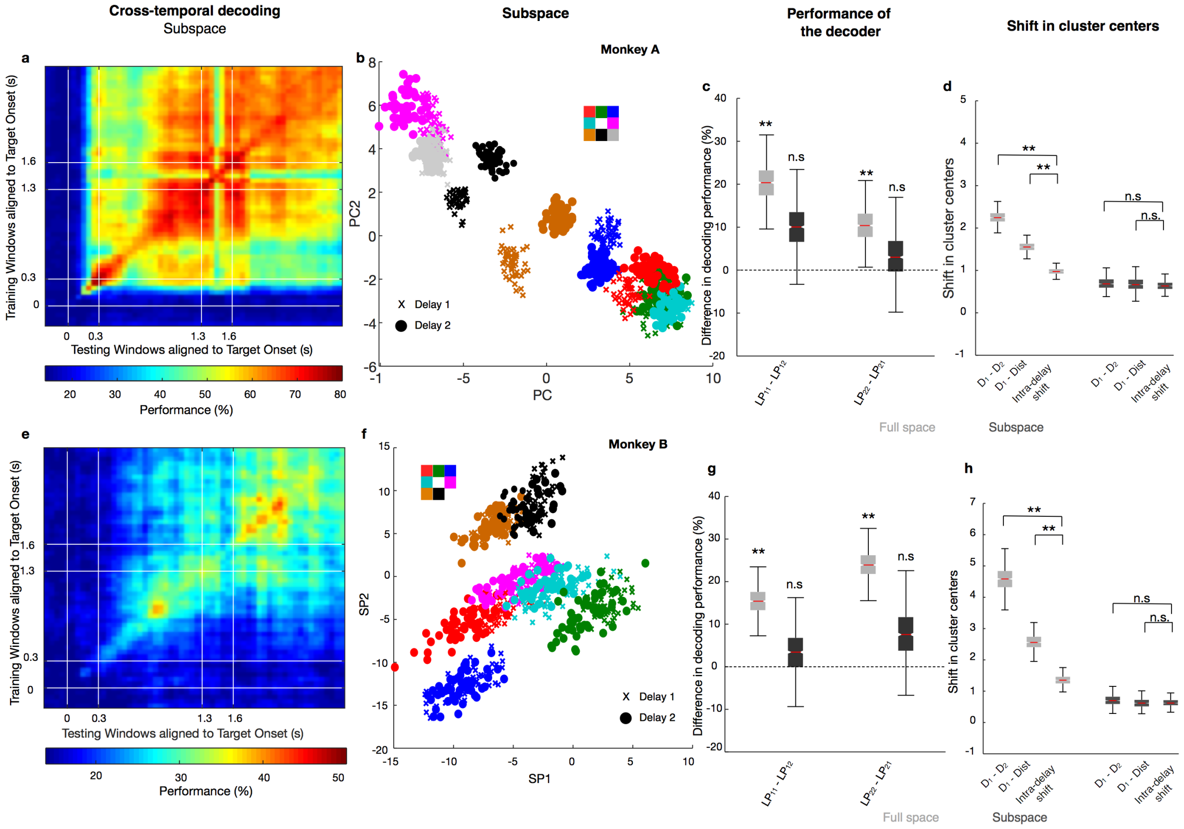

Supplementary Figure 1. Identification of a subspace in individual monkeys. a, Heat map showing the cross-temporal population-decoding performance after the population responses were projected into the subspace. White lines indicate target presentation (0–0.3 s) and distractor presentation (1.3–1.6 s). **b,** Delay 1 (plotted using crosses) and Delay 2 (plotted using squares) responses after projection onto the top 2 dimensions of the subspace. Points for different target locations are color-coded according to the color scheme shown in the top left. **c,** The difference in decoding performance when a decoder that was trained on Delay 1 responses was tested on Delay 1 (LP_{11}) or Delay 2 (LP_{12}) responses are shown in the light gray box-plot labelled LP_{11} - LP_{12} (P < 0.001), and vice versa (light gray box-plot labelled LP_{22} - LP_{21}, P < 0.001). The equivalent performance differences in the subspace are shown in the dark gray box-plots (LP_{11} - LP_{12}, P ≈ 0.17; LP_{22} - LP_{21}, P ≈ 0.41). **d,** The shift in cluster centers from Delay 1 to Delay 2 (labeled D1 - D2, full space: P < 0.001, g = 8.19; subspace: P ≈ 0.92) averaged across target locations, from Delay 1 to the distractor presentation period (D1 – Dist, subspace: P < 0.001, g = 2.38; fullspace: P ≈ 0.94), and the intra-delay shifts in both Delays 1 and 2 (intra-delay) are shown in the full space (light gray box-plot), and in the subspace (dark gray box-plot). **e-h,** same as a-d for Monkey B. Fullspace: LP_{11} - LP_{12} (P < 0.001); LP_{22} - LP_{21} (P < 0.001); subspace: LP_{11} - LP_{12} (P ≈ 0.47); LP_{22} - LP_{21} (P ≈ 0.53). Fullspace: D1 - D2 (P < 0.001, g = 6.49); D1 – Dist (P< 0.001, g = 2.37); subspace: D1 - D2 (P ≈ 0.97); D1 – Dist (P ≈ 0.96). The red lines in boxplot denote the median of the distribution and the whiskers represent the 2.5th and 97.5th percentile. In all panels, asterisks (**) denote significance (i.e., 95th percentile range of the distribution did not overlap with zero, or the 95th percentile range of two distributions did not overlap). The p-values were computed using a non-parametric permutation test described in the methods.

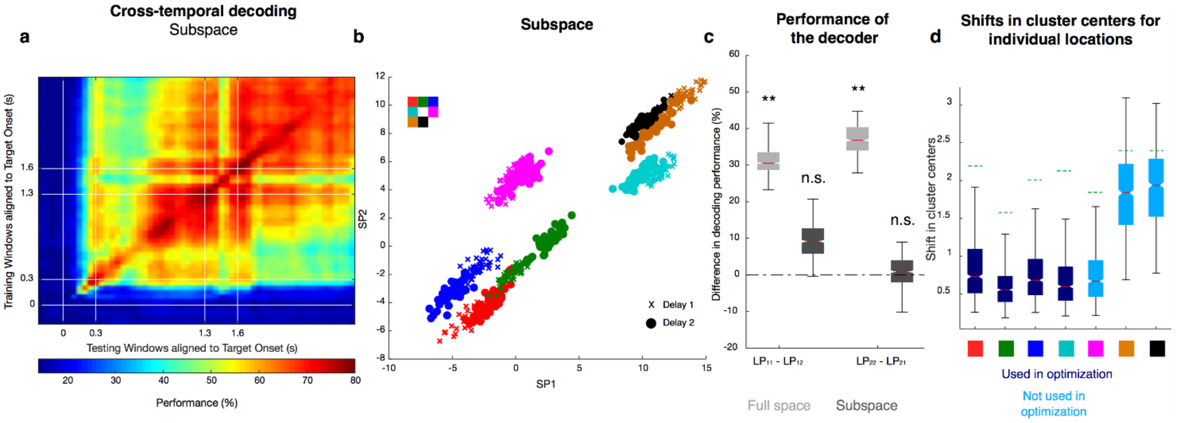

Supplementary Figure 2. Optimized subspace using 4 locations also produced a time-invariant subspace. a, Heat map showing the cross-temporal population-decoding performance after the population responses were projected onto the optimized subspace found using 4 target locations (locations shown in red, green, blue and cyan in the inset in fig b). **b,** Delay 1 (plotted using crosses) and Delay 2 (plotted using circle) responses after projection into the top 2 PCs of the subspace. Points for different target locations are color-coded according to the color scheme shown in the top left. **c,** Box-plots showing the performance of the full space (gray bars) and subspace (black bars) decoders ($P < 0.001$ for LP11 - LP12 and LP22 - LP21 in the full space, $P \approx 0.09$ for LP11 - LP12 and $P \approx 0.49$ for LP22 - LP21 in the subspace). **d,** Box-plots showing the shifts in cluster centers between delay 1 and delay 2 for individual locations. The green dotted line represents the 97.5th percentile of the intra-delay shift. The locations denoted in dark blue are used in the optimization ($P \approx$ 0.85, 0.89, 0.91, 0.92, 0.94, 0.82 and 0.83 for the box-plots from left to right.). The red lines denote the median of the distribution and the whiskers represent the 2.5th and 97.5th percentile of the distribution. P-values mentioned here are computed using a non-parametric permutation test.

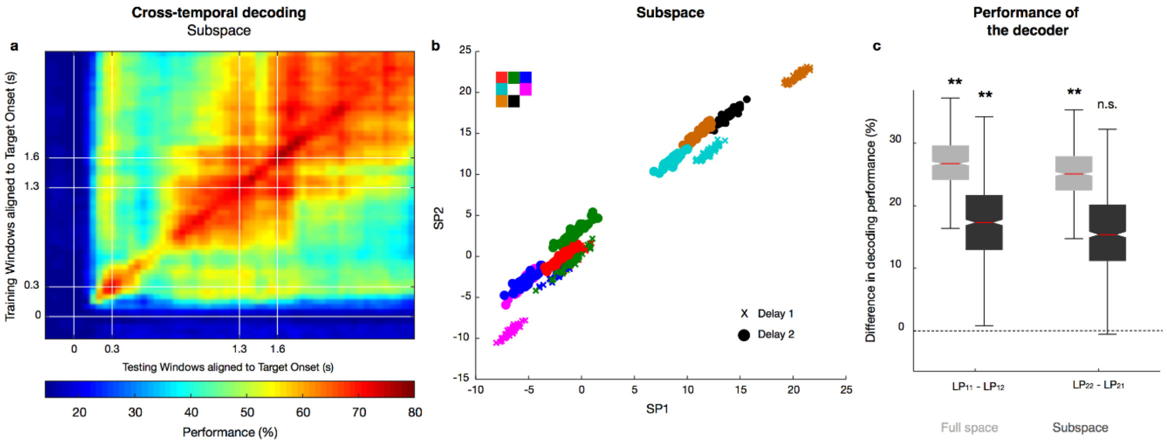

Supplementary Figure 3. **Optimized subspace using Target and Delay 2 periods does not lead to a time-invariant subspace. a,** Heat map showing the cross-temporal population-decoding performance after the population responses were projected onto the optimized subspace found using Target and Delay 2 periods. **b,** Delay 1 (plotted using crosses) and Delay 2 (plotted using squares) responses after projection into the top 2 PCs of the subspace. Points for different target locations are color-coded according to the color scheme shown in the top left. **c,** Box-plots showing the performance of the full space (gray bars) and subspace (black bars) decoders ($P < 0.001$ for LP11 - LP12 in full space and the subspace, $P < 0.001$ for LP22 - LP21 in full space and $P \approx 0.17$ for LP22 - LP21 in the subspace). The red line in the boxplots represent the median of the distribution and the whiskers represent the 2.5^{th} and 97.5^{th} percentile of the distribution. P-values mentioned here are computed using a non-parametric permutation test.

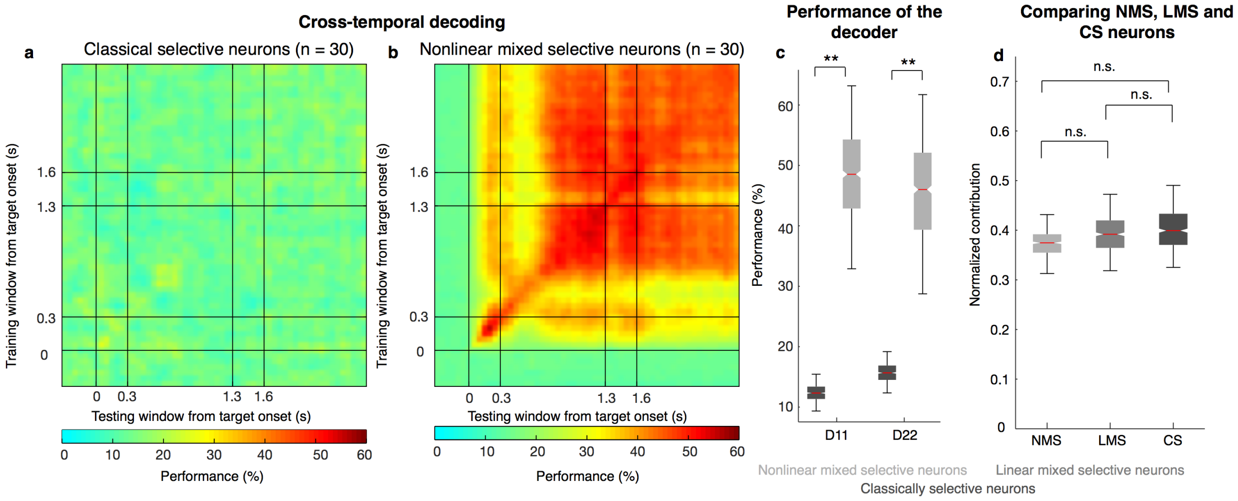

Supplementary Figure 4. **Subspace decoding using subpopulations of LPFC neurons.** **a,** Heatmap showing subspace decoding using only CS neurons. **b,** Heatmap showing subspace decoding using only NMS neurons. **c,** Box-plots showing the performance of the subspace decoder for CS (black box-plots) and NMS neurons (gray box-plots) in Delays 1 and 2. The performance for the CS neurons were significantly lower than those for the NMS neurons (D_{11}: $P < 0.001$ $g = 10.28$ D_{22}: $P < 0.001$ $g = 8.71$). **d,** Contribution of different types of neurons (including neurons with linear mixed-selectivity, LMS) to the subspace shown in Figure 2b. There were no significant differences between the 3 distributions ($P \approx 0.85$ for NMS and, LMS, $P \approx 0.81$ for LMS and, CS and $P \approx 0.91$ for NMS and CS). The red line in the boxplots represent the median of the distribution and the whiskers represent the 2.5th and 97.5th percentile of the distribution. P-values mentioned here are computed using a non-parametric permutation test.

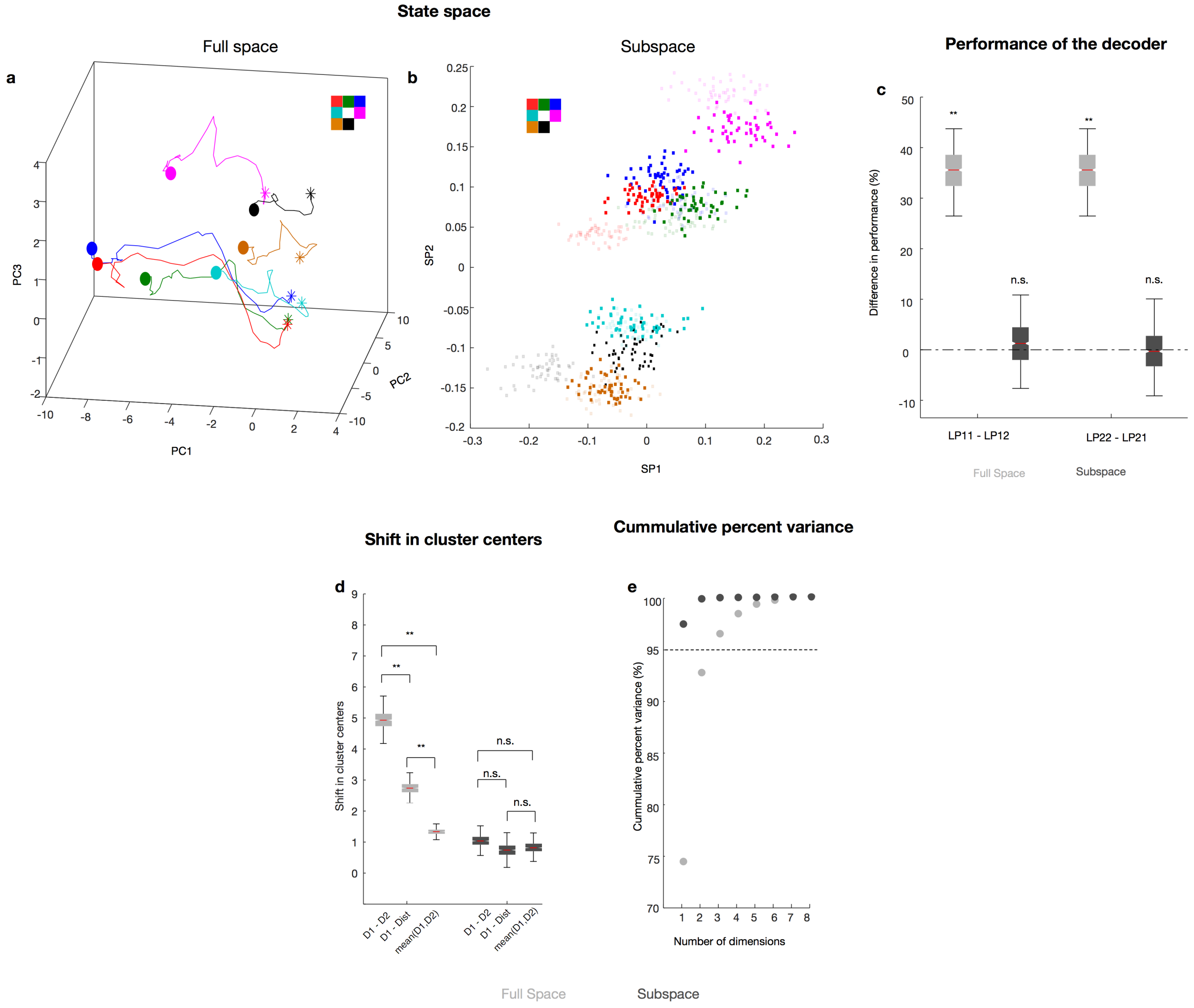

Supplementary Figure 5. **Characterization of the full space and subspace found using only NMS neurons. a,** Responses of the NMS neurons when projected onto the first three principal components (PC) of the combined Delay 1 and Delay 2 response space. The responses for different target locations are color-coded using the color scheme shown in the top left. The trajectories illustrate the evolution of the responses from 500 ms before the end of Delay 1 (square), distractor onset (first triangle), distractor offset (second triangle), and the end of Delay 2 (circle). The trajectories of only 4 of the 7 target locations are shown here for clarity. **b,** Delay 1 (plotted using crosses) and Delay 2 (plotted using squares) responses after projection into the top 2 PCs of the subspace. Points for different target locations are color-coded according to the color scheme shown in the top left. **c,** The difference in decoding performance when a decoder that was trained on Delay 1 responses was tested on Delay 1 (LP_{11}) or Delay 2 (LP_{12}) responses are shown in the gray box-plot labelled LP_{11} - LP_{12}, and vice versa (gray box-plot labelled LP_{22} - LP_{21}, $P < 0.001$ for LP_{11} - LP_{12} and LP_{22} - LP_{21}). The equivalent performance differences in the subspace are shown in the black box-plots ($P \approx 0.71$ for LP_{11} - LP_{12} and $P \approx 0.57$ for LP_{22} - LP_{21}). **d,** The shift in cluster centers from Delay 1 to Delay 2 (labeled D1 - D2) averaged across target locations, from Delay 1 to the distractor presentation period (D1 - Dist), and the intra-delay shifts in both Delays 1 and 2 (intra-delay) are shown in the full space (gray box-plot, $P < 0.001$ for D1 - D2 and D1 - Dist, $g = 20.75$ for D1 - D2 and 16.54 for D1 - Dist), and in the subspace (black box-plot, $P \approx 0.93$ for D1 - D2 and $P \approx 0.95$ for D1 - D2). **e,** The cumulative explained variance is plotted as a function of the

number of PCs for the full space (plotted in gray) and the subspace (plotted in black). The red line in boxplots represent the median of the distribution and the whiskers represent the 2.5^{th} and 97.5^{th} percentile of the distribution. P-values mentioned here are computed using a non-parametric permutation test.

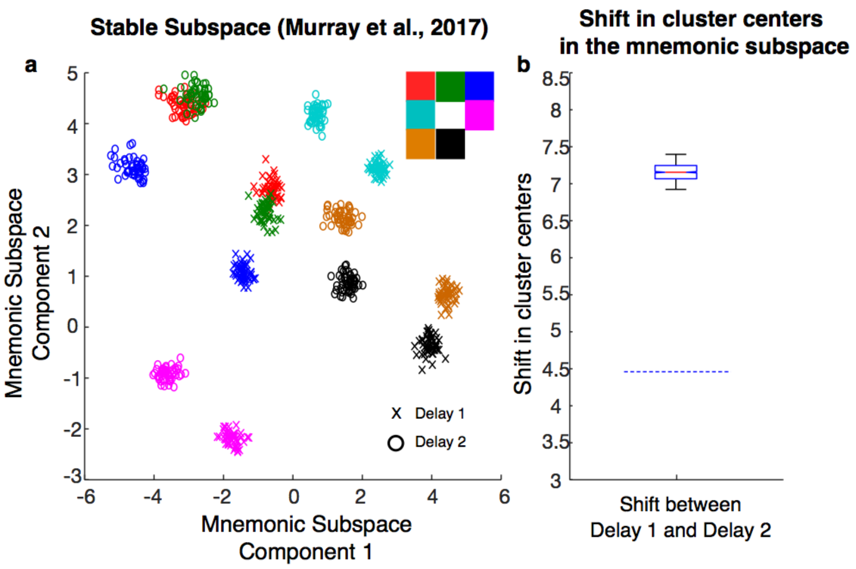

Supplementary Figure 6. Mnemonic Subspace. a, Responses of LPFC neurons when projected onto the first two principal components of the mnemonic subspace. The responses for different target locations are color-coded using the color scheme shown in the top right. Responses in Delay 1 are plotted using crosses (x), while responses in Delay 2 are plotted using circles (o). **b,** The shift in cluster centers from Delay 1 to Delay 2 (averaged across target locations) are shown for the mnemonic subspace (computed over 58 components, which accounted for 95% of the variance). The dotted lines show the 97.5th percentile of the intra-delay shifts averaged across target locations. The inter-delay shifts were significantly different from the intra-delay shifts ($P < 0.001$, $g = 31.62$). The red line in boxplots represent the median of the distribution and the whiskers represent the 2.5^{th} and 97.5^{th} percentile of the distribution. P-values mentioned here are computed using a non-parametric permutation test.

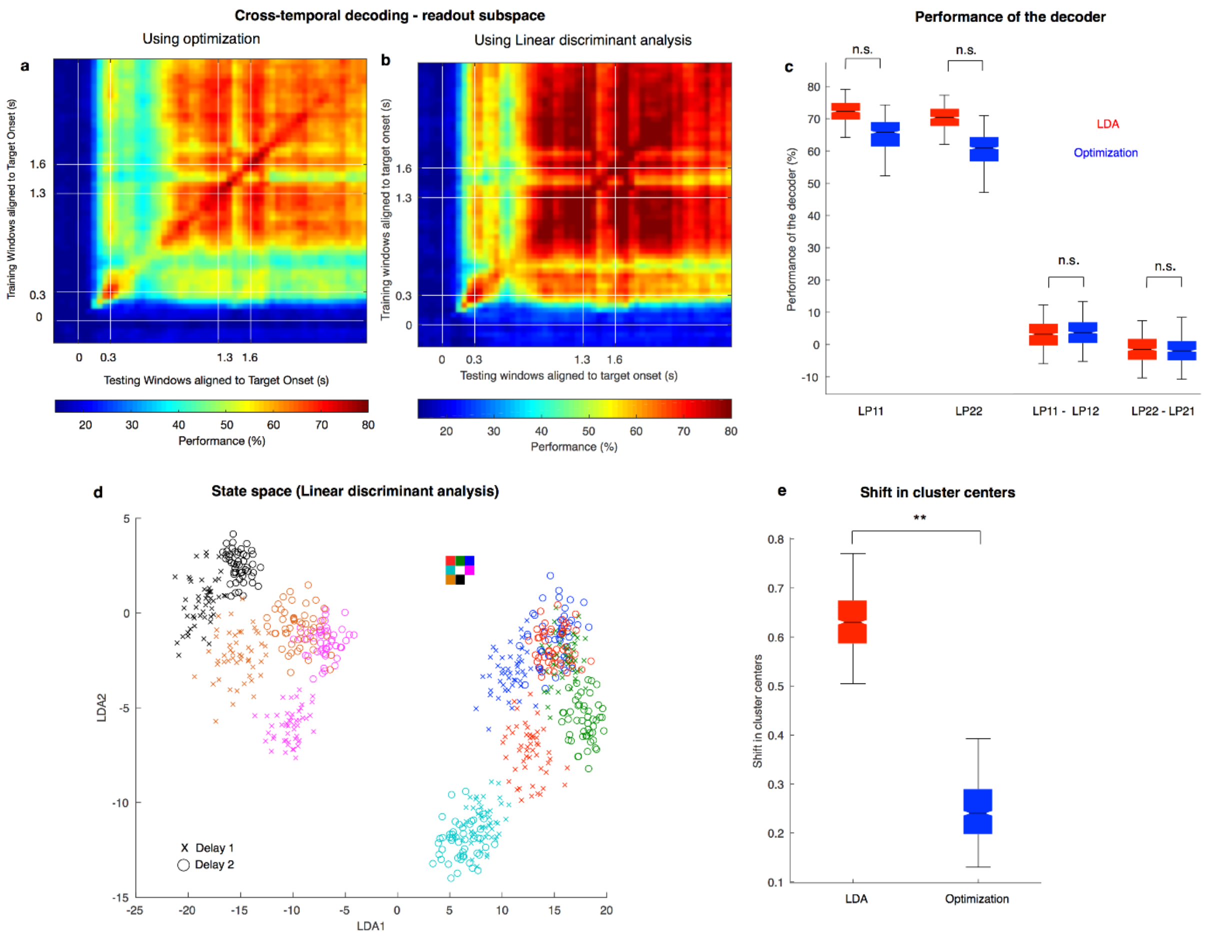

Supplementary Figure 7. **LDA Subspace. a**, Heat map showing the cross-temporal population-decoding performance after the population responses were projected onto the optimized subspace (shown previously in Fig. 2c). **b,** Heat map showing the cross-temporal population-decoding performance after the population responses were projected onto the LDA subspace. **c,** The decoding performance in the LDA subspace was not significantly different from the optimized subspace (LP_{11}: $P \approx 0.13$; LP_{22}: $P \approx 0.14$; LP_{11} - LP_{12}: $P \approx 0.99$; LP_{22} - LP_{21}: $P \approx 0.98$). **d,** Delay 1 (plotted using crosses, x) and Delay 2 (plotted using circles, o) responses after projecting into 2 of the LDA boundaries. Points for different target locations are color-coded according to the color scheme shown in the top right. **e,** The shift in cluster centers from Delay 1 to Delay 2 (averaged across target locations) are shown for the LDA subspace (plotted in red, computed over 8 components, which accounted for 95% of the variance) and the optimized subspace (plotted in blue, shown previously in Fig. 2e). The shift in the LDA subspace was significantly larger than that in the optimized subspace ($P \approx 0.01$, $g = 9.07$). The red line in boxplots represent the median of the distribution and the whiskers represent the 2.5th and 97.5th percentile of the distribution. P-values mentioned here are computed using a non-parametric permutation test.

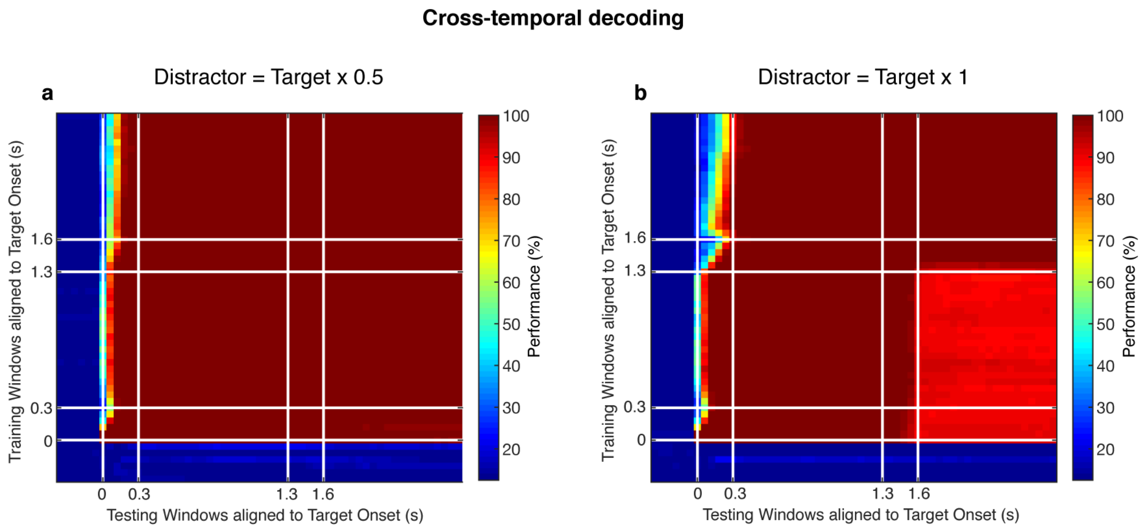

Supplementary Figure 8. **Target and distractor activity levels in the bump attractor model. a**, Heat map showing the lack of code-morphing when the activity level of the distractor inputs was half that of the target. **b**, Heat map showing the lack of code-morphing in the cross-temporal population-decoding performance of the model in the full space when the activity level of the distractor inputs was the same as the target.

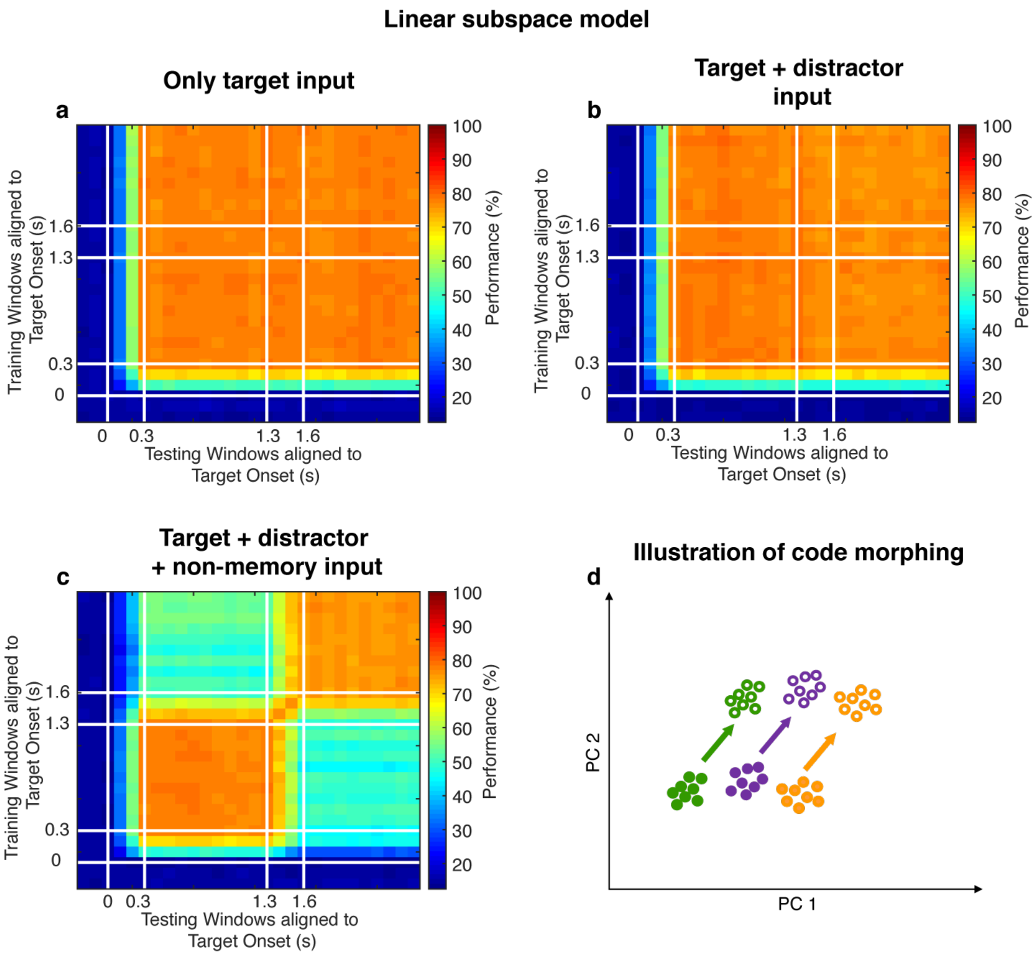

Supplementary Figure 9. **Linear subspace model. a**, The input weights for target locations were specified as the stable eigenvectors of the recurrent weight matrix (see Methods), and thus the cross-temporal decoding exhibited stability when only target information was present. **b**, The addition of a distractor input, with lower magnitude than that for target input, did not lead to code morphing. **c**, With the addition of a non-memory input in the distractor period, the linear subspace model displayed code morphing, but it was driven by neurons with linear mixed selectivity (LMS, as described in Methods), which made up $92.7 \pm 3.1\%$ of the whole population, while neurons with nonlinear mixed selectivity (NMS) only made up $6.6 \pm 2.9\%$ of the whole population. This was different from the neural data, in which we found 54% of the selective neurons to be neurons with NMS, and 24% were neurons with LMS. Similarly, the bump attractor model was made up of 91 ± 1.6% neurons with NMS, and 5.4 ± 2.3% neurons with LMS. **d**, Illustration of code morphing driven by the non-memory input. Different colors represent different target clusters; closed circles are Delay 1 activity, open circles are Delay 2 activity. As the same non-memory input was added for all the target locations, the translation for all the target clusters in state space would be identical. That meant that for each single neuron, the change from Delay 1 to Delay 2 would be the same for all target locations, and thus the neuron will be classified as a neuron with LMS. Please see the Model section in the Methods for a more extended discussion of the differences between the linear subspace model and the bump attractor model.

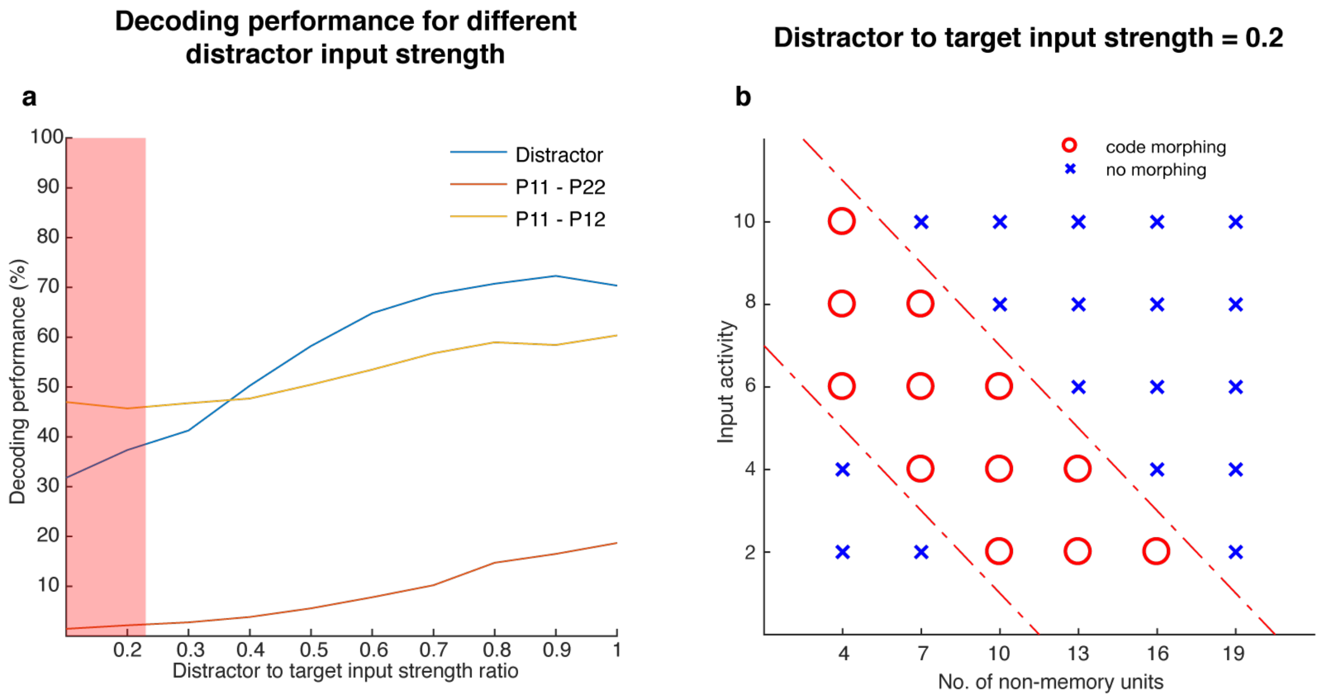

Supplementary Figure 10. **Parameters in the bump attractor model**. **a,** The decoding performance in the model for the distractor location (plotted in blue) is shown for different distractor activity levels relative to the target activity level. The difference in performance between LP_{11} and LP_{22}, and between LP_{11} and LP_{12}, are plotted in red and yellow, respectively. The red bar indicates a range of distractor activity levels that replicated the lower distractor decoding performance compared to the target decoding performance. **b,** We tested different pairs of combinations of different numbers of non-memory units, *n*, and activity level of the non-memory inputs, *s*, when the distractor to target input strength was 0.2, and found that the pairs that successfully replicated code-morphing exhibited an anti-correlation between *n* and *s*.

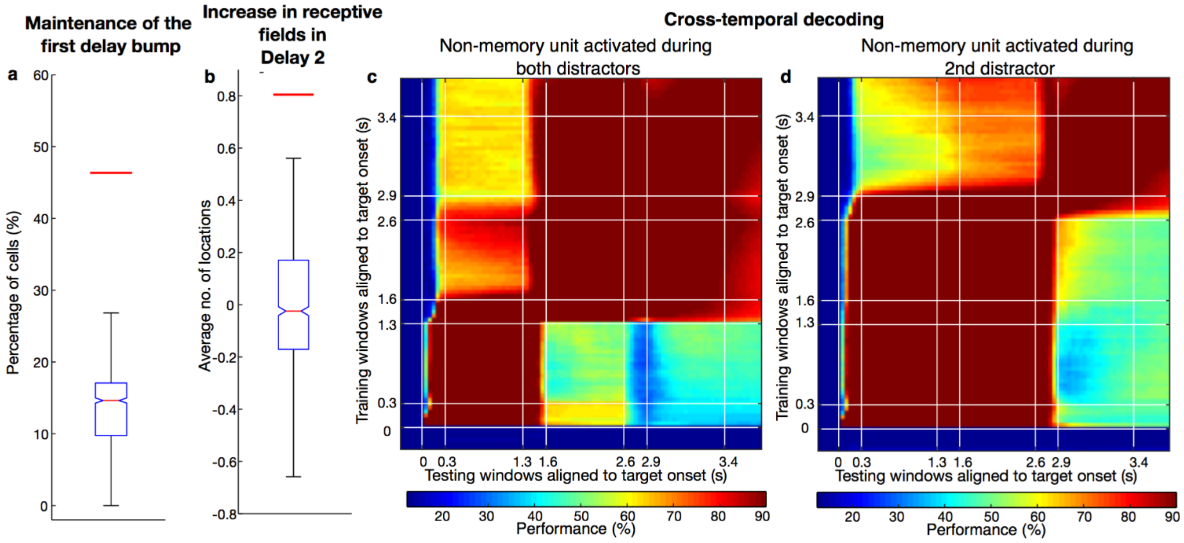

Supplementary Figure 11. **Predictions of the bump attractor model. a,** The percentage of NMS cells that maintained its selectivity in Delay 1 into Delay 2 (characterized simply by a lack of change in the most responsive location in both delays) is shown in the red horizontal line, while the box-plot illustrate the 5th and 95th percentile of the null distribution, which shuffled the responses for different locations within each delay ($P < 0.001$, $g = 5.85$). **b,** The number of responsive locations for each neuron increased in Delay 2 compared to Delay 1 ($P < 0.001$, $g = 3.26$). This corresponded to the addition of the non-memory bump to the target location bump in Delay 2. The red horizontal line shows the average increase in locations, while the box-plots illustrate the 5th and 95th percentile of the null distribution, which shuffled responses for different locations between the two delays. **c,** In a simulation with 2 distractors presented, the non-memory input (assumed to be an ascending input) was activated during both distractor presentations, so code morphing only occurred after the first distractor was presented. **d,** In a simulation with 2 distractors presented, the non-memory input (assumed to be movement preparation or reward expectation) was only activated during the second distractor presentation, so code morphing only occurred after the second distractor was presented. The red line of the boxplots represent the median of the distribution and the whiskers represent the 2.5^{th} and 97.5^{th} percentile of the distribution. P-values mentioned here are computed using a non-parametric permutation test.
