# Supplementary material for: Time-invariant working memory representations in the presence of code-morphing in the lateral prefrontal cortex
Source: Nat Commun. 2019 Nov 1;10:4995. doi: 10.1038/s41467-019-12841-y (PMC6825148; doi:10.1038/s41467-019-12841-y)
Supplement: Supplementary file 3 — Description of Additional Supplementary Files [file 41467_2019_12841_MOESM3_ESM.pdf]

Description of Additional Supplementary Files

File Name: Supplementary Movie 1
Description: LPFC and FEF state space movie
